# Prostate cancer burden in South Asia: A systematic analysis of global burden of disease data (1990–2021)

**DOI:** 10.1111/iju.15641

**Published:** 2024-12-13

**Authors:** Vijay Kumar, Diptismita Jena, Quazi Syed Zahiruddin, R. Roopashree, Mandeep Kaur, Manish Srivastava, Amit Barwal, G. V. Siva Prasad, Pranchal Rajput, Rukshar Syed, Gajendra Sharma, Sunil Kumar, Nagavalli Chilakam, Ganesh Bushi, Hassan Basri Jahubar Sathik, Rachana Mehta, Sanjit Sah, Muhammed Shabil, Abhay M. Gaidhane, Hashem Abu Serhan

**Affiliations:** ^1^ Global Center for Evidence Synthesis Chandigarh India; ^2^ Center for Global Health Research, Saveetha Medical College and Hospital, Saveetha Institute of Medical and Technical Sciences Saveetha University Chennai India; ^3^ School of Pharmaceutical Sciences Lovely Professional University Phagwara India; ^4^ South Asia Infant Feeding Research Network (SAIFRN), Division of Evidence Synthesis, Global Consortium of Public Health and Research Datta Meghe Institute of Higher Education Wardha India; ^5^ Department of Chemistry and Biochemistry, School of Sciences JAIN (Deemed to be University) Bangalore Karnataka India; ^6^ Department of Allied Healthcare and Sciences Vivekananda Global University Jaipur Rajasthan India; ^7^ Department of Endocrinology NIMS University Jaipur India; ^8^ Chandigarh Pharmacy College Chandigarh Group of College Jhanjeri, Mohali Punjab India; ^9^ Department of Chemistry Raghu Engineering College Visakhapatnam Andhra Pradesh India; ^10^ School of Applied and Life Sciences, Division of Research and Innovation Uttaranchal University Dehradun India; ^11^ IES Institute of Pharmacy IES University Bhopal Madhya Pradesh India; ^12^ New Delhi Institute of Management Tughlakabad Institutional Area New Delhi India; ^13^ Department of Microbiology Graphic Era (Deemed to be University) Clement Town Dehradun India; ^14^ Noida Institute of Engineering and Technology (Pharmacy Institute) Greater Noida India; ^15^ University of Cyberjaya Persiaran Bestari, Cyber 11 Cyberjaya Selangor Malaysia; ^16^ Clinical Microbiology RDC, Manav Rachna International Institute of Research and Studies Faridabad Haryana India; ^17^ Department of Paediatrics, Dr. D. Y. Patil Medical College, Hospital and Research Centre Dr. D. Y. Patil Vidyapeeth Pune Maharashtra India; ^18^ Department of Public Health Dentistry, Dr. D.Y. Patil Dental College and Hospital Dr. D.Y. Patil Vidyapeeth Pune Maharashtra India; ^19^ University Center for Research and Development Chandigarh University Mohali Punjab India; ^20^ Medical Laboratories Techniques Department AL‐Mustaqbal University Hillah Babil Iraq; ^21^ Jawaharlal Nehru Medical College, and Global Health Academy, School of Epidemiology and Public Health Datta Meghe Institute of Higher Education Wardha India; ^22^ Department of Ophthalmology Hamad Medical Corporation Doha Qatar

**Keywords:** Asian populations, Genital neoplasms, Global burden of disease, Prostatic neoplasms

## Abstract

**Objectives:**

The objectives of this study were to analyze trends in prostate cancer incidence, incidence, mortality, and disability‐adjusted life years (DALYs) from 1990 to 2021 via data from the Global Burden of Disease (GBD) study in South Asia. Additionally, the study projects future prostate cancer incidence rates up to 2031 to inform public health interventions in South Asia.

**Methods:**

Data covering South Asian countries such as Bangladesh, Bhutan, India, Nepal, and Pakistan were obtained from the GBD 2021 portal. Age‐standardized rates (ASRs) for prostate cancer metrics, including incidence (ASIR), prevalence (ASPR), mortality (ASMR), and DALYs (ASDR), were analyzed via joinpoint and ARIMA modeling techniques. Geographic variations in ASRs were mapped via QGIS software.

**Results:**

The prostate cancer ASIR, ASPR, and ASDR significantly increased from 1990 to 2021, particularly among individuals aged 60–65 years. The highest incidence and mortality rates were observed in Pakistan. The total percentage change in incidence in India was the highest at 61%. Projections indicate a continued rise in prostate cancer incidence, with South Asia's ASIR expected to reach 9.34 per 100 000 by 2031.

**Conclusions:**

The growing burden of prostate cancer in South Asia highlights the need for enhanced screening programs, public awareness, and healthcare infrastructure improvements. Without intervention, the increasing incidence and mortality rates could strain healthcare resources, emphasizing the urgency of region‐specific public health strategies.

## INTRODUCTION

Prostate cancer is one of the most prevalent malignancies affecting men globally, ranking as the second most frequently diagnosed cancer and a significant cause of cancer‐related mortality in 2012.^1^ In 2020 alone, approximately 1 414 259 new cases and 375 304 deaths were attributed to this disease worldwide, highlighting its substantial impact on global health.^2^ While historically considered a disease of Western nations, the incidence of prostate cancer has been steadily increasing across Asia, including South Asia.^1^, ^3^ This increase has been linked to several factors, such as aging, lifestyle changes, and improved detection methods such as prostate‐specific antigen (PSA) testing.^1^, ^4^, ^5^ Despite its growing incidence, prostate cancer remains underreported in many parts of Asia, partly owing to immature cancer registry systems and limited access to screening technologies.^6^ Initiative like the United in Fight against Prostate cancer registries aim to enhance data on diagnosis, treatment, and outcomes.^7^ Despite these efforts, challenges such as regional disparities and insufficient data collection persist, complicating comprehensive reporting. Furthermore, survival rates appear higher in Asian populations, suggesting underreporting of early‐stage cases, particularly in less developed areas.^8^, ^9^

In South Asia, the burden of prostate cancer is becoming more apparent, especially as awareness of the disease and screening methods remains low.^10^ Studies have demonstrated a stark lack of knowledge and public awareness regarding prostate cancer in the region. Many men are not aware of the PSA test, and screening programs are not yet well established.^10^ The region's socioeconomic disparities exacerbate the problem, as healthcare infrastructure struggles to keep pace with the increasing number of cases.

The incidence of prostate cancer varies significantly across Asian countries. In South Asia, the rate is relatively low compared with that in Western nations, but it has been increasing in recent years. The mortality‐to‐incidence ratio in South Asia, however, remains high, reflecting the challenges associated with early detection and timely intervention.^1^ As the population continues to age and Western dietary habits become more prevalent, the burden of prostate cancer in the region is projected to increase significantly in the coming decades.^11^

Despite the increase in reported cases, there remains a significant gap in comprehensive cancer surveillance and detailed epidemiological studies across South Asia. The lack of robust data is a barrier to understanding the full impact and scope of prostate cancer in the region. This analysis aims to bridge that gap by presenting a detailed review of the burden of prostate cancer in South Asia from 1990 to 2021, utilizing data from the GBD study. This study comprehensively examines the regional differences in incidence, prevalence, mortality rates, and DALYs associated with prostate cancer while also projecting future trends in disease burden across South Asia. These findings highlight the critical need for targeted health interventions and the development of well‐informed public health strategies to address the escalating threat of prostate cancer effectively in the region.

## METHODS

The GBD Study 2021 provides a comprehensive assessment of health trends worldwide, utilizing an extensive array of 328 938 data sources to offer insights into health disparities influenced by age, sex, location, and socioeconomic factors. The methodology employed involves a rigorous analysis of secondary data sources, including censuses, population registries, vital registration systems, hospital and health insurance data, surveys, disease registries, morbidity notifications, and both published and unpublished literature. This approach ensures the inclusion of diverse data types to create comparable, high‐quality estimates across various geographies and time frames. The study prioritizes methodological transparency, requiring thorough documentation for each data source to aid researchers in understanding and utilizing the data effectively. Advanced statistical and epidemiological techniques are applied to address data gaps and increase the robustness of health estimates.^12^, ^13^

In the present study, we obtained data from the GBDx portal (https://vizhub.healthdata.org/gbd‐results/?params=gbd‐api‐2021‐public/) for prostate cancer in South Asia, including countries such as Bangladesh, Bhutan, Nepal, Pakistan, and India. Data were retrieved on ASIR, ASPR, ASDR, and ASMR among males from 1990 to 2021.

The agewise distribution of prostate cancer in South Asia was visualized via a bar plot created in Microsoft Excel. The plot covered plots aged 30 years and above, with 5‐year intervals up to 75+ years.

The joinpoint analysis was conducted via Joinpoint software (version 5.2.0) of the National Cancer Institute. Joinpoint software analyzes trends, such as cancer rates, by fitting the simplest possible model within user‐specified limits on the number of joinpoints. In this study, we applied the best‐fitting model suggested by the software for each health metric analyzed, including prevalence, incidence, mortality, and DALY rates.^14^

We projected the ASIRs of prostate cancer in South Asian countries up to 2031 via GBD data from 1990 to 2021. For this projection, the ARIMA model was chosen and employed via R software and the “Forecast” package.^15^ Data were made stationary via second‐order differencing in the ASIR of prostate cancer. We specified the ARIMA model by determining the three critical parameters—p, d, and q—after rendering the time series stationary and examining autocorrelations. The parameters represent the order of the autoregressive part (p), the degree of differencing (d), and the order of the moving average part (q), collectively denoted as ARIMA (p, d, q). We utilized autocorrelation function (ACF) and partial autocorrelation function (PACF) plots to identify suitable values for p and q, as these plots assist in discerning the nature of data autocorrelation—guiding the choice of AR or MA terms.^16^, ^17^

Boundary maps for the ASIRs and ASMRs of prostate cancer patients in South Asia were created via QGIS software. The rate data were combined with the shapefile of South Asia in this software, and color coding was applied on the basis of the rates.

## RESULTS

**FIGURE 1:**
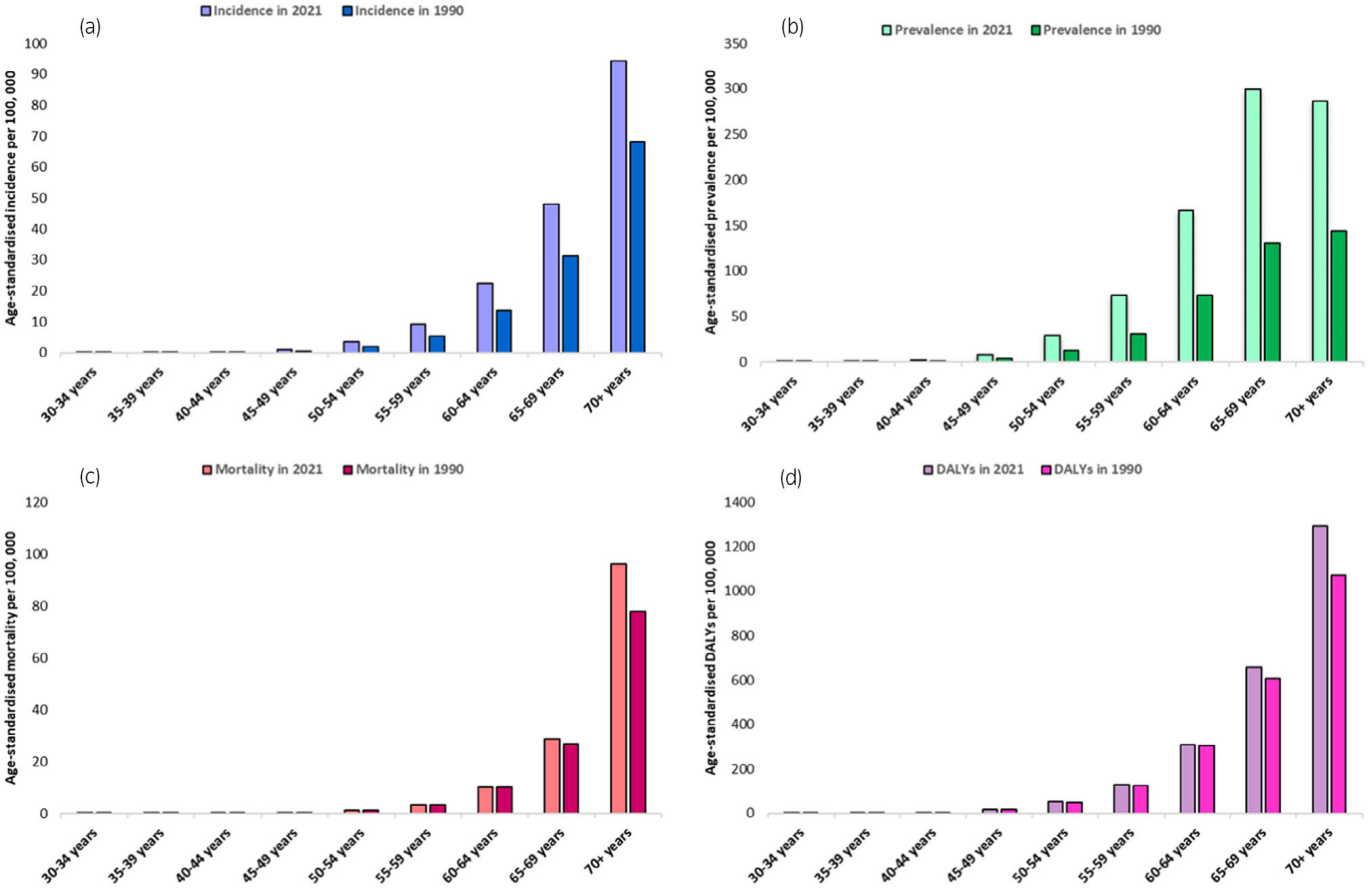
Age‐distribution of age‐standardized rates of prostate cancer in South Asia.

The groups aged 65–69 years and 70+ years reported the highest percentages of all the ASRs included in the analysis. The rates of all health metrics increased from 1990 to 2021. Interestingly, the prevalence rate almost more than doubled in 2021 compared with 1990 in all the age groups, with the highest rate occurring in the 60–65 years group (Figure 1).

**FIGURE 2:**
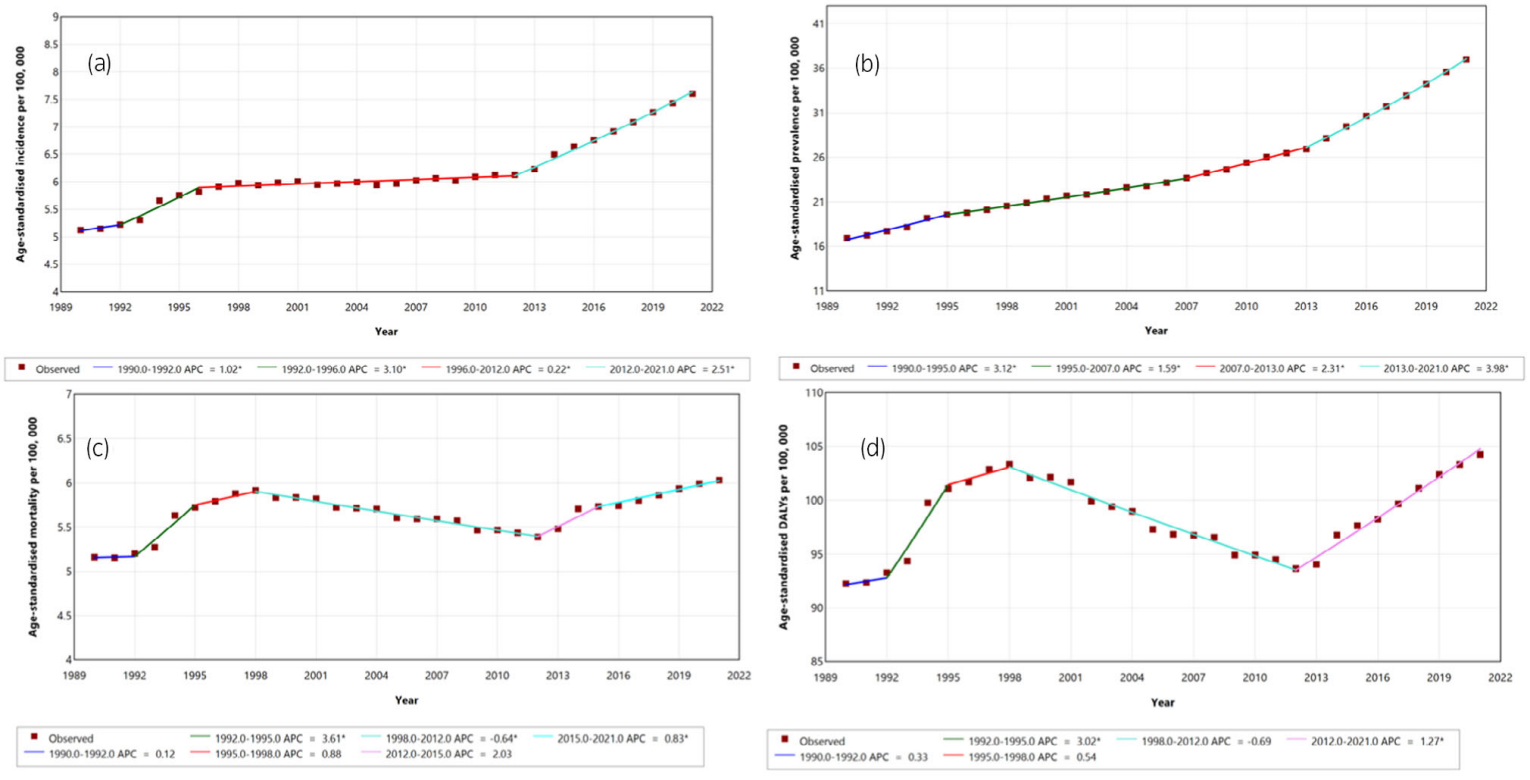
Trends of age‐standardized rates of prostate cancer in South Asia from 1990 to 2021. (a) Joinpoint of age‐standardized incidence rate; (b) Joinpoint of age‐standardized prevalence rate; (c) Joinpoint of age‐standardized mortality rate; (c) Joinpoint of age‐standardized DALYs rate. *Indicates that the annual percentage change (APC) is significantly different from the zero at the alpha = 0.05 level.

The joinpoint analysis revealed the highest annual percentage change (APC) in the ASIR between 1992 and 1996, followed by the years between 2012 and 2021, with values of 3.10 and 2.51, respectively (Figure 2a). Between 2012 and 2021, the highest ASPR change was noted, followed by 1990–1995, with APCs of 3.98 and 3.12, respectively (Figure 2b). Figure 2c shows the irregular trends for the ASMR, although the highest APC was noted from 1992 to 1995, with an APC value of 3.61. For the ASDR, the highest APC was observed between 1992 and 1995, with a value of 3.02 (Figure 2d). Although the trend is increasing, from 1998 to 2012, the trends show a decline in the ASMR and ASDR and approximately stable trends in the ASIR.

Table 1 shows that between 1990 and 2021, the average annual percentage change (AAPC) in age‐standardized rates for prostate cancer in South Asia showed varying trends across different health metrics. The ASIR increased with an AAPC of 1.3 (95% CI: 1.25–1.38), whereas the prevalence rate significantly increased, with an AAPC of 2.59 (95% CI: 2.55–2.64). Mortality and DALYs also experienced slight increases, with mortality showing an AAPC of 0.5 (95% CI: 0.44–0.56) and DALYs increasing by 0.41 (95% CI: 0.37–0.46).

**TABLE 1:** Average annual percentage change of age‐standardized rates of prostate cancer in South Asia from 1990 to 2021.

Health metrics	Year	Average annual percentage change (95% CI)
Age‐standardized incidence rate	1990 to 2021	1.3* (1.25 to 1.38)
Age‐standardized prevalence rate	1990 to 2021	2.59* (2.55 to 2.64)
Age‐standardized mortality rate	1990 to 2021	0.5* (0.44 to 0.56)
Age‐standardized DALYs rate	1990 to 2021	0.41* (0.37 to 0.46)

Abbreviation: AAPC: DALYs, disability‐adjusted life years.

* Indicates AAPC is significantly different from zero at the alpha = 0.05 level.

**FIGURE 3:**
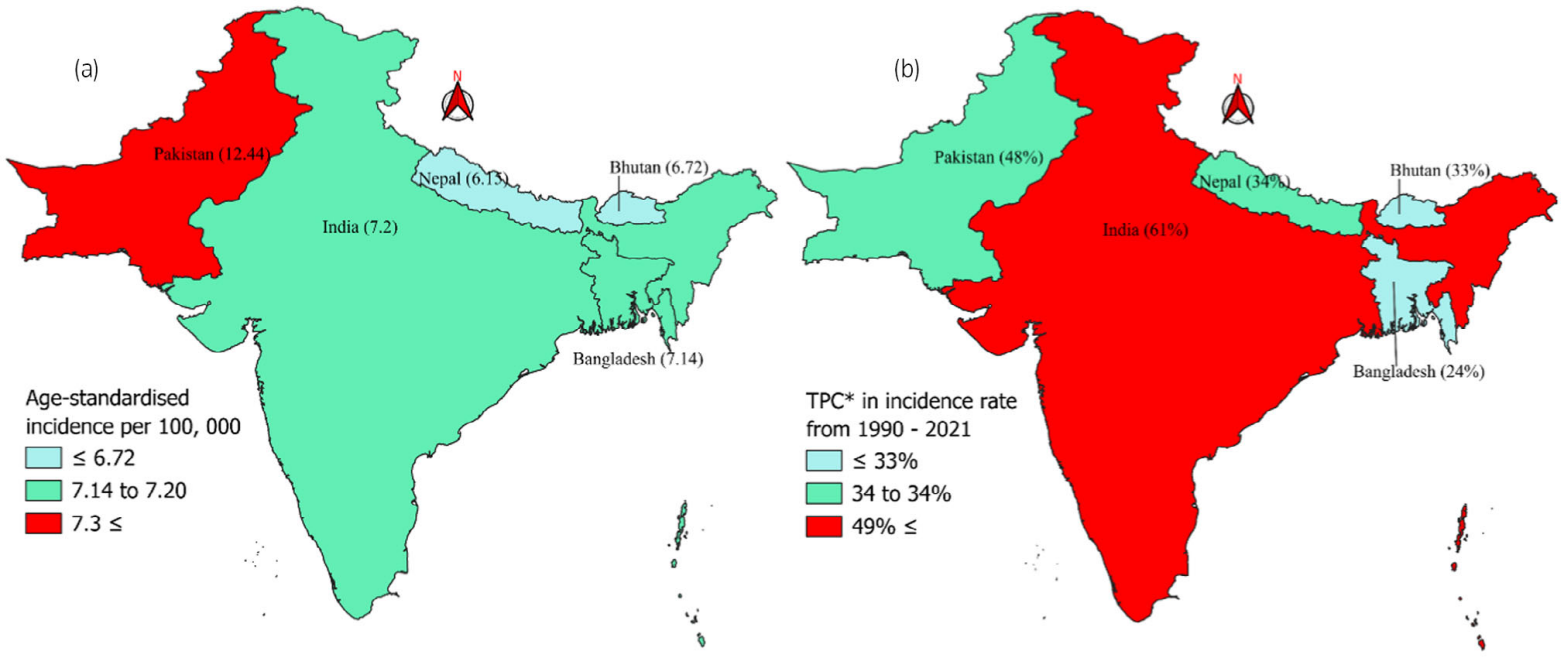
Map illustrating age‐standardized incidence rate differences and total percentage change (TPC) in South Asia from 1990 to 2021. (a) Age‐standardized incidence per 100 000 prostate cancer in South Asia. (b) *Total percentage change (TPC) in incidence rate of prostate cancer from 1990 to 2021.

**FIGURE 4:**
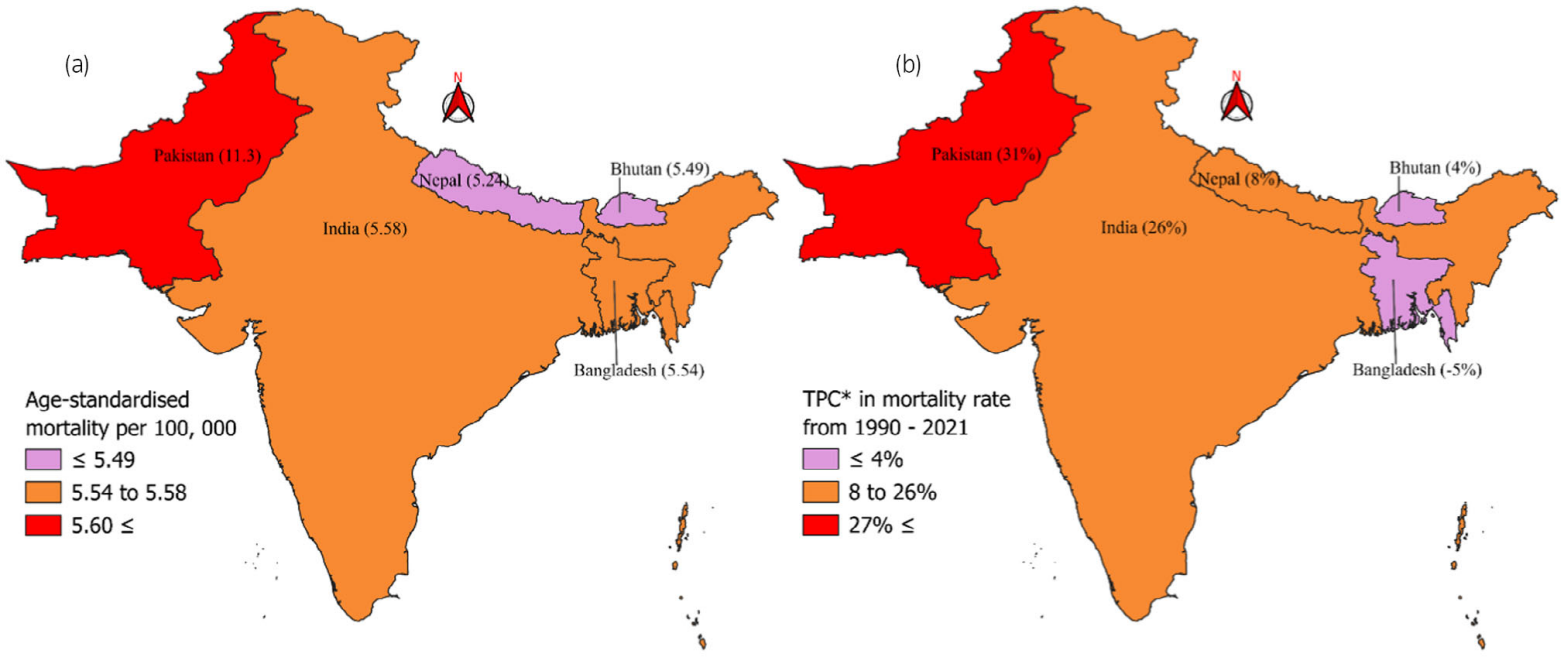
Map illustrating age‐standardized mortality rate differences and total percentage change (TPC) in South Asia from 1990 to 2021. (a) Age‐standardized mortality per 100 000 prostate cancer in South Asia. (b) *Total percentage change (TPC) in mortality rate of prostate cancer 1990 to 2021.

Figure 3a shows Pakistan as the country with the highest incidence rate in 2021 among South Asian countries, with an ASIR of 12.44 per 100 000. Countries other than Pakistan presented similar ASIRs, ranging from 6.15 to 7.2 per 100 000. Although Pakistan had the highest ASIR in 2021, India reported the highest TPC from 1990 to 2021 for ASIR in South Asia, with a TPC of 61%, followed by Pakistan (48%). Other South Asian countries ranged from 24% (Bangladesh) to 34% (Nepal) (Figure 3b). Similar results were found for the ASMR, as Pakistan reported the highest mortality rate in 2021, with an ASMR of 11.3 per 100 000. Other South Asian countries reported similar mortality rates in 2021, with ASMRs ranging from 5.24 to 5.58 per 100 000 people (Figure 4a). Pakistan presented a TPC of 31% from 1990 to 2021, which was the highest among the other South Asian countries, followed by India (24%). Bangladesh is the only southern Asian country with a decreasing TPC from 1990 to 2021, with a value of −5%.

The TPC in the ASPR and ASDR for prostate cancer from 1990 to 2021 in South Asia shows significant variations across countries (Table 2). South Asia as a whole experienced a 118% increase in prevalence and a 13% increase in DALYs. Bangladesh reported a 107% increase in prevalence but an 11% reduction in DALYs. Bhutan exhibited a 116% increase in prevalence, with a marginal 1% increase in DALYs. India experienced the greatest increase in prevalence at 133%, with DALYs increasing by 19%. Nepal prevalence rose by 110%, whereas DALYs increased by 7%. Pakistan, although showing a lower prevalence increase of 88%, recorded a 30% increase in DALYs.

**TABLE 2:** Country wise total percentage change in age‐standardized rates for prevalence and DALYs of prostate cancer from 1990 to 2021 in South Asian countries.

States	1990 age‐standardized rates		2021 age‐standardized rates		TPC from 1990 to 2021	
	Prevalence (U.I)	DALYs (U.I)	Prevalence (U.I)	DALYs (U.I)	Prevalence (U.I)	DALYs (U.I)
South Asia	16.93 (20.17 to 12.54)	5.12 (6.31 to 3.63)	36.99 (49.02 to 30.23)	7.6 (10.42 to 6.26)	118 (183 to 73)	13 (49 to −14)
Bangladesh	17.67 (24.72 to 11.09)	106.57 (152.52 to 64.26)	36.56 (66.36 to 19.53)	94.83 (172.23 to 51.69)	107 (213 to 24)	−11 (34 to −45)
Bhutan	14.86 (23.19 to 8.53)	91.53 (144.36 to 50.16)	32.06 (58.24 to 18.33)	92.34 (169.92 to 53.34)	116 (234 to 28)	1 (60 to −40)
India	15.38 (18.67 to 10.66)	80.68 (101.67 to 50.95)	35.85 (46.93 to 28.25)	96.37 (130.72 to 76.67)	133 (217 to 80)	19 (74 to −11)
Nepal	12.9 (18.9 to 8.3)	83.61 (127.07 to 50.69)	27.04 (46.7 to 16.86)	89.06 (157.88 to 55.04)	110 (221 to 35)	7 (70 to −33)
Pakistan	26.81 (36.21 to 19.64)	151.17 (217.21 to 107.61)	50.42 (69.96 to 35.53)	196.53 (277.26 to 134.57)	88 (193 to 29)	30 (107 to −14)

*Note*: TPC = $\left(\frac{\text{Rate in}\;2021-\text{Rate in}\;1990}{\text{Rate in}\;1990}\right)*100$.

Abbreviations: DALYs, disability‐adjusted life years; TPC, annual percentage change; UI, uncertainty interval.

**FIGURE 5:**
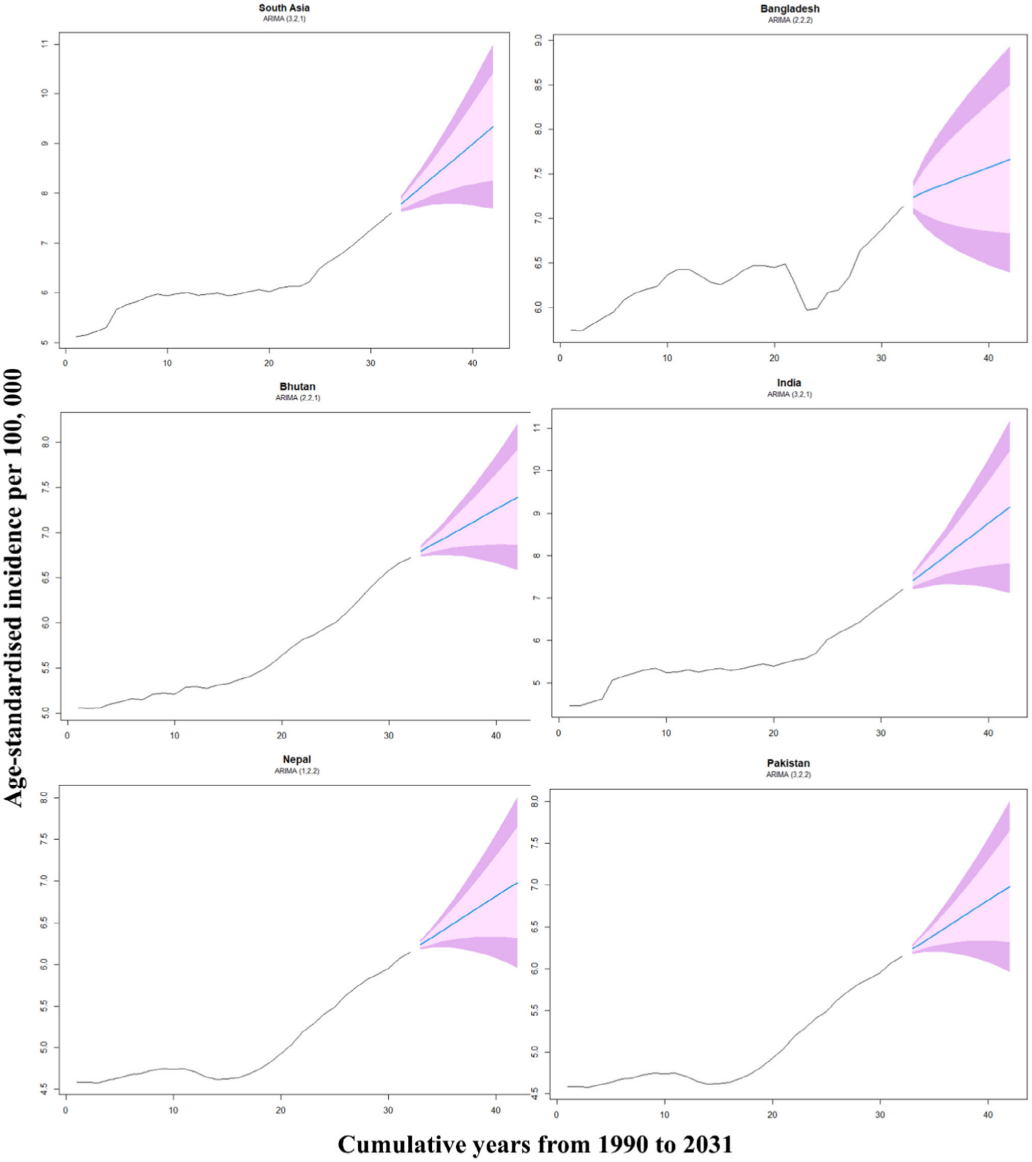
Forecasting age‐standardized incidence rates of prostate cancer in South Asian countries (up to 2031) using the ARIMA model and global burden of disease data (1990–2021).

Figure 5 shows the increasing incidence rates of ASIRs in all South Asian countries. The ASIR for South Asia is projected to increase from 7.78 per 100 000 in 2022 to 9.34 per 100 000 by 2031. Within the region, Bangladesh is expected to experience a modest increase in ASIR from 7.24 in 2022 to 7.66 in 2031. Similarly, Bhutan's ASIR is forecasted to increase from 6.79 to 7.39 over the same period. Notably, India's ASIR is projected to increase significantly from 7.41 in 2022 to 9.15 in 2031, marking the highest anticipated growth among South Asian nations. Additionally, both Nepal and Pakistan are expected to experience increases in their ASIRs, with rates rising from 6.24 to 6.99 by 2031 for both countries (Table 3).

**TABLE 3:** Prostate cancer incidence forecast in South Asia (2022–2031): projections based on global burden of disease data (1990–2021).

Year	South Asia ARIMA (3, 2, 1)	Bangladesh ARIMA (2, 2, 2)	Bhutan ARIMA (2, 2, 1)	India ARIMA (3, 2, 1)	Nepal ARIMA (3, 2, 2)	Pakistan ARIMA (0, 2, 2)
	Predicted ASIR (95% CI)	Predicted ASIR (95% CI)	Predicted ASIR (95% CI)	Predicted ASIR (95% CI)	Predicted ASIR (95% CI)	Predicted ASIR (95% CI)
2022	7.78 (7.62 to 7.94)	7.24 (7.05 to 7.42)	6.79 (6.73 to 6.86)	7.41 (7.21 to 7.61)	6.24 (6.18 to 6.29)	6.24 (6.18 to 6.29)
2023	7.95 (7.67 to 8.24)	7.29 (6.91 to 7.68)	6.86 (6.75 to 6.98)	7.6 (7.25 to 7.95)	6.32 (6.2 to 6.43)	6.32 (6.2 to 6.43)
2024	8.13 (7.73 to 8.52)	7.34 (6.8 to 7.89)	6.92 (6.75 to 7.1)	7.79 (7.29 to 8.28)	6.4 (6.21 to 6.6)	6.4 (6.21 to 6.6)
2025	8.3 (7.77 to 8.83)	7.39 (6.71 to 8.07)	6.99 (6.75 to 7.24)	7.98 (7.33 to 8.63)	6.48 (6.2 to 6.77)	6.48 (6.2 to 6.77)
2026	8.47 (7.78 to 9.17)	7.44 (6.64 to 8.23)	7.06 (6.74 to 7.38)	8.18 (7.32 to 9.04)	6.57 (6.18 to 6.96)	6.57 (6.18 to 6.96)
2027	8.65 (7.78 to 9.51)	7.48 (6.58 to 8.38)	7.13 (6.72 to 7.54)	8.37 (7.31 to 9.44)	6.65 (6.16 to 7.15)	6.65 (6.16 to 7.15)
2028	8.82 (7.77 to 9.86)	7.53 (6.53 to 8.53)	7.19 (6.69 to 7.7)	8.57 (7.28 to 9.85)	6.74 (6.12 to 7.35)	6.74 (6.12 to 7.35)
2029	8.99 (7.76 to 10.23)	7.57 (6.48 to 8.67)	7.26 (6.66 to 7.86)	8.76 (7.24 to 10.28)	6.82 (6.07 to 7.56)	6.82 (6.07 to 7.56)
2030	9.17 (7.72 to 10.61)	7.62 (6.43 to 8.8)	7.33 (6.63 to 8.03)	8.96 (7.18 to 10.73)	6.9 (6.02 to 7.78)	6.9 (6.02 to 7.78)
2031	9.34 (7.68 to 10.99)	7.66 (6.39 to 8.94)	7.39 (6.58 to 8.21)	9.15 (7.12 to 11.18)	6.99 (5.96 to 8.01)	6.99 (5.96 to 8.01)

Abbreviations: ARIMA (Autoregressive Integrated Moving Average) is a statistical method used to forecast future disease trends based on historical data; ASIR, age‐standardized incidence rate per 100 000.

## DISCUSSION

The current study provides a comprehensive overview of the prostate cancer burden in South Asia, revealing both temporal trends and future projections from 1990 to 2021. These findings underscore the critical and growing impact of prostate cancer in the region, with increasing incidence, prevalence, and DALYs. This growing burden is expected to continue, as indicated by our projections up to 2031, emphasizing the urgency for public health interventions tailored to South Asia's unique demographic, socioeconomic, and healthcare challenges.

One of the most notable findings in this analysis is the significant increase in the incidence and prevalence of prostate cancer in South Asia. From 1990 to 2021, the region experienced an alarming increase in the ASPR across all age groups, particularly in the 60–65 years age group. Similarly, the ASIR markedly increased, with Pakistan reporting the highest ASIR in 2021. This trend is consistent with previous research that indicates the increasing burden of prostate cancer in Asia as a whole, driven by aging populations, lifestyle changes, and improvements in diagnostic capabilities. The increasing incidence of prostate cancer in Pakistan, with one study showing a rise from 3.88% to 5.80% between 2000 and 2023, is attributed to factors such as aging, obesity, smoking, and genetic predispositions such as the TMPRSS2 gene polymorphism.^18^, ^19^ Higher prevalence rates are observed in regions such as Khyber Pakhtunkhwa and Punjab, likely because of regional lifestyle and environmental factors. Limited awareness and inadequate screening programs further contribute to late diagnoses.^20^ In line with findings from Kimura et al. (2018), our study affirms that lifestyle changes, including dietary shifts and increased adoption of Western habits, play crucial roles in the increasing incidence of prostate cancer in South Asia.^1^

This study highlights considerable geographic variation in the prostate cancer burden across South Asia, with Pakistan showing the highest ASIR and ASMR in 2021. Interestingly, India presented the highest TPC in the ASIR from 1990 to 2021, reflecting the rapid increase in disease burden in a country with a large population base. Similar findings have been reported, with rising incidence rates particularly noted in urban areas such as Delhi, Kamrup Urban, and Mumbai.^21^ Factors contributing to this rise include genetic predispositions, lifestyle changes such as diet and smoking, and late‐stage diagnoses.^22^ A large percentage of prostate cancer cases in India are diagnosed at advanced stages, with 43% reaching the distant metastatic stage.^21^ Bangladesh, on the other hand, was the only country that demonstrated a decrease in mortality over the same period. These variations may be attributed to differences in healthcare infrastructure, public awareness, and the availability of screening programs, such as PSA testing.

Projections for prostate cancer incidence up to 2031 indicate a continued rise across all South Asian countries, with the region's ASIR expected to reach 9.34 per 100 000 by 2031. India, in particular, is projected to experience the most significant increase in ASIR, likely driven by its large and aging population. This anticipated rise underscores the urgent need for increased efforts in early detection, effective management strategies, and healthcare infrastructure improvements to address the growing burden of prostate cancer in the country.

Improving the survival rate of patients with prostate cancer can be achieved through timely interventions and personalized treatment strategies. For advanced prostate cancer, studies have shown that sequencing treatments, such as the use of abiraterone followed by enzalutamide (AA → ENZ), improve outcomes and are more cost‐effective, which is particularly beneficial for low‐ and middle‐income countries, including those in South Asia.^23^ Early detection through active surveillance for low‐risk patients helps delay aggressive treatments, preserving quality of life while ensuring close monitoring.^24^, ^25^ Additionally, new therapies such as enzalutamide, abiraterone, and cabozantinib offer further benefits for advanced cases.^23^ Increased awareness, improved screening programs, and personalized treatment plans can significantly increase survival rates and reduce unnecessary treatments.

This study has several limitations that should be acknowledged. First, the reliance on secondary data sources may limit the accuracy of estimates in certain countries where cancer registries are underdeveloped or incomplete. Second, the absence of uniform screening practices across South Asia introduces variability in the reported incidence and prevalence rates. Finally, our projections are based on past trends and do not account for potential future healthcare interventions or policy changes that could alter the trajectory of prostate cancer in the region.

In conclusion, the findings from this study provide critical insights into the growing burden of prostate cancer in South Asia, highlighting the need for urgent action to address rising incidence and mortality rates. Public health strategies that prioritize early detection, improved awareness, and enhanced healthcare infrastructure are essential for mitigating the impact of prostate cancer in the region. Without timely intervention, the disease burden is likely to escalate, further straining already limited healthcare resources.

## AUTHOR CONTRIBUTIONS

**Vijay Kumar:** Conceptualization; formal analysis; methodology; writing – review and editing; writing – original draft; visualization; software. **Diptismita Jena:** Conceptualization; methodology; visualization; software; writing – review and editing; writing – original draft. **Quazi Syed Zahiruddin:** Conceptualization; writing – original draft; writing – review and editing; software; visualization; methodology. **R. Roopashree:** Software; visualization; writing – review and editing. **Mandeep Kaur:** Writing – original draft; methodology. **Manish Srivastava:** Software; visualization. **Amit Barwal:** Writing – original draft. **G. V. Siva Prasad:** Writing – review and editing. **Pranchal Rajput:** Writing – review and editing. **Rukshar Syed:** Writing – review and editing; writing – original draft. **Gajendra Sharma:** Writing – review and editing. **Sunil Kumar:** Validation; writing – review and editing. **Nagavalli Chilakam:** Writing – review and editing. **Ganesh Bushi:** Software; visualization. **Hassan Basri Jahubar Sathik:** Writing – original draft. **Rachana Mehta:** Methodology. **Sanjit Sah:** Writing – review and editing; writing – original draft. **Muhammed Shabil:** Writing – review and editing. **Abhay M. Gaidhane:** Writing – review and editing; software; visualization. **Hashem Abu Serhan:** Writing – original draft; writing – review and editing; supervision.

## CONFLICT OF INTEREST STATEMENT

No conflict of interest declared.

## APPROVAL OF THE RESEARCH PROTOCOL BY AN INSTITUTIONAL REVIEWER BOARD

Not applicable.

## INFORMED CONSENT

Not applicable.

## REGISTRY AND THE REGISTRATION NO. OF THE STUDY/TRIAL

Not applicable.

## ANIMAL STUDIES

Not applicable.

## FUNDING INFORMATION

No funding.

## Data Availability

Available at Institute for Health Metrics and Evaluation (IHME) site (https://vizhub.healthdata.org/gbd‐results/).
